# Perception of Dental House Officers regarding Endodontic File Separation during Endodontic Treatment

**DOI:** 10.1155/2023/1044541

**Published:** 2023-02-16

**Authors:** Manahil Maqbool, Syeda Sobia Masood Tirmazi, Asma Shakoor, Zunaira Akram, Rutaba Nazir, Arham Nawaz Chohan, Mohmed Isaqali Karobari, Tahir Yusuf Noorani

**Affiliations:** ^1^Paediatric Dentistry Unit, School of Dental Sciences, Universiti Sains Malaysia, Health Campus, 16150 Kubang Kerian, Kota Bharu, Kelantan, Malaysia; ^2^Operative Dentistry Department, CMH-Lahore Medical College and Institute of Dentistry (NUMS), Lahore, Pakistan; ^3^Community & Preventive Dentistry Department, CMH-Lahore Medical College and Institute of Dentistry (NUMS), Lahore, Pakistan; ^4^Department of Oral Biology, UHS-University of Health Sciences, Lahore, Pakistan; ^5^Pakistan Atomic Energy Commission General Hospital, Islamabad, Pakistan; ^6^Department of Pediatric Dentistry, Institute of Dentistry, CMH Lahore Medical College National University of Medical Sciences, Lahore, Pakistan; ^7^Department of Restorative Dentistry & Endodontics, Faculty of Dentistry, University of Puthisastra, Phnom Penh 12211, Cambodia; ^8^Conservative Dentistry & Endodontics, Saveetha Dental College & Hospitals, Saveetha Institute of Medical and Technical Sciences University, Chennai, Tamil Nadu 600077, India; ^9^Conservative Dentistry Unit, School of Dental Sciences, Universiti Sains Malaysia, Health Campus, 16150 Kubang Kerian, Kota Bharu, Kelantan, Malaysia

## Abstract

**Background:**

Despite of having improved endodontic file designs as well as the reinforced metal alloy file structure, intracanal endodontic file separation (EFS) is still a very problematic and worrisome dental incident, which usually occurs without any visible signs of permanent deformation. Further, there have been conflicting reports regarding the clinical significance of leaving separated files within root canals.

**Aims:**

The aim of this study was to look into the current perceptions and awareness about file separation during endodontic treatment among the dental house officers (DHOs).

**Materials and Methods:**

A novel validated questionnaire comprising of 15 close-ended questions was distributed anonymously via Google Forms through email to 1100 DHOs across Pakistan. The questionnaire consisted of two parts: the first component (Section I) collected demographic data and the second component (Section II) investigated the causes of EFS during root canal treatment. Following the completion of socioeconomic information, including age and gender, the DHOs were asked to answer a few questions about the various reasons for endodontic instrument fracture.

**Results:**

A total of 800 responses were recorded, with an effective rate of 72.8%. The majority of the DHOs (*p* value < 0.001) perceived that endodontic instrument fracture occurred in the posterior (61.5%) and apical third of the canal (50.5%) and in older permanent dentition (67.3%), possibly due to patient anxiety (62%). Better choice of instrument (61.15%), operators' experience (95.3%), knowledge (87.5%), and proper root canal cleaning (91.1%) are believed to be the vital steps in reducing endodontic file separation/fracture. Furthermore, majority of them (*p* value < 0.001) perceived that stainless steel was a superior alloy for filing instruments. Manual files tend to be more prone to fractures due to repeated use than rotary files.

**Conclusion:**

This study demonstrated that young DHOs had adequate knowledge and awareness regarding the potential predisposing factors and handling techniques for EFS. This study thereby provides an evaluating tool to access the insights of the current perceptions and awareness of DHOs concerning EFS.

## 1. Introduction

Endodontics is thought to be one of the most difficult branches of dentistry due to its microhandling as well as the extremely diverse methodologies and protocols. Endodontic treatment is becoming more popular owing to rising life expectancy, people's desire to keep their natural teeth, and patients' awareness of the benefits of preserving their teeth rather than extracting them [1]. This highlights the importance of dental students being skilled enough and having a firm grasp on the fundamentals of endodontic procedures to be able to perform them on their own [2].

Considering the anatomical differences between teeth, providing distinct instrumentation techniques, aseptic debridement, and a satisfactory obturation are just a few of the essential steps to ensuring the success of a root canal treatment [3]. However, despite the improved file designs as well as the reinforced metal alloy file structure, intracanal endodontic file separation (EFS) is still a very problematic and worrisome dental incident [4]. It usually occurs without any visible signs of permanent deformation. Moreover, there have been conflicting reports regarding the clinical significance of leaving separated files within root canals. The possible factors that contribute to the EFS are operator proficiency, understanding root anatomy, choice of metals, and multiple usage of endodontic instruments [4]. The EFS is a worrisome event that hinders efficient cleaning and shaping of the root canal system, thereby affecting the treatment and prognosis. Eventually, the treated case could fail. Therefore, the understanding and knowledge of the mechanisms and contributing factors leading to EFS are necessary, especially for newly graduated dentists, also known as dental house officers (DHOs), in order to reduce the incidence of EFS in future root canal treatments.

Previous studies indicate many procedural errors that are common during an endodontic procedure, such as voids, ledge formation, overfilling, broken instruments, and underfilling [5–7]. The percentage incidence of an intracanal instrument fracture ranges extensively between 0.28% and 16.20% [3, 8–10]. Furthermore, earlier questionnaire-based studies provide ample data on the quality of endodontic treatment obtained from undergraduate students, but there is hardly any data that reflects students' perception and awareness towards endodontics with corresponding self-confidence in delivering various steps of root canal treatment, especially regarding EFS [11]. Moreover, the frequency and prevalence of EFS is described differently across the literature, making it more difficult to determine [12–14]. The focus of these studies is normally confounded by factors such as tooth location, associated operative difficulties, and operator experience. As a result, the literature accounting of studies that report EFS is widely comprehensive [15, 16]. The management of EFS is challenging, and the success rate of EFS is very low, even among the specialists. We believe the knowledge of EFS would instill confidence among DHOs.

This study focuses on the routine struggles endured by DHOs while performing endodontic procedures, as well as the underlying complications that they may routinely encounter based on different perceptions. The study's null hypothesis is based on the fact that DHOs' knowledge of EFS does not influence the oral health services they provide to patients. We aimed to evaluate the current perceptions and awareness of DHOs regarding file separation during endodontic treatment in dental institutes across Pakistan, in order to provide a more comfortable and stress-free clinical environment for operators, which would help improve the standard of oral health services provided to the community, as well as to establish an evaluating survey that can be deemed useful for DHOs around the world in contemplating their awareness and management skills of EFS.

## 2. Methodology

### 2.1. Study Design

This cross-sectional study was carried out over a six-month period, ranging from March 2021 to August 2021, among DHOs across 10 different dental institutes in Pakistan. Data was collected using a newly validated questionnaire for Evaluation of Perception: Part 1 (Appendix 1). The ethical approval was obtained on February 28, 2021, from the Institutional Review Board of CMH Lahore Medical College and Institute of Dentistry (case number: 596/ERC/CMH/LMC).

### 2.2. Inclusion and Exclusion Criteria

All DHOs who had completed their compulsory clinical rotation after graduation in the Department of Endodontics while completing at least 25 root canal treatment (RCT) cases were included in this study, whereas all DHOs who had not yet completed their compulsory clinical rotation after graduation in the department of Endodontics, as well as DHOs who had not completed 25 RCT cases, were excluded from this study.

### 2.3. Sampling Method

**Figure 1:**
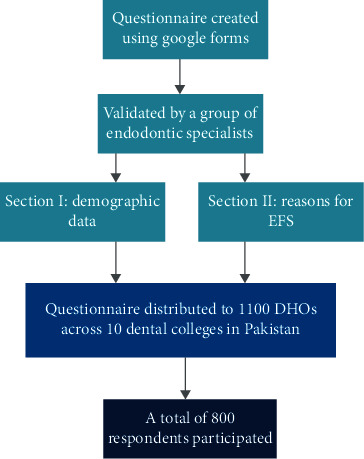
Study flow and sampling method illustration.

Initially, a panel of five experts reviewed and suggested changes to the questionnaire for face validation, validating whether the questionnaire is relevant and appropriate for measuring the study objectives. The developed questionnaire was then pilot tested on 60 participants. To ensure consistency, the response data was cleaned by entering it into a spreadsheet and comparing individuals' responses to positive- and negative-phrased questions. The questionnaire was revised at this point, and a well-structured questionnaire was created based on the expert's suggestions (Supplementary Information (available here)). The questionnaire gathered demographic data and the reasons for endodontic instrument fracture during root canal treatment. The questionnaire was distributed widely to all respondents online via various social media platforms using Google Forms. The questionnaire link was sent to a total of 1100 DHOs across the ten randomly selected dental colleges in Pakistan, and they were filled out voluntarily and anonymously online by the graduating DHOs of different respective colleges. The purpose of the study was explained at the beginning of the questionnaire. Informed consent was obtained. The DHOs were told that their participation was voluntary and their response would not have an effect on their grading of educational performance in their respective colleges. The confidentiality of their identities and the right to participate were also clarified. Subsequently, socioeconomic information was filled in, including age and gender; also, the DHOs were also asked to fill in a few questions regarding different reasons for EFS.  Figure 1 illustrates the sampling method of the study.

### 2.4. Statistical Analysis

The descriptive analysis of the data obtained via an online questionnaire was conducted using the Social Science Statistical Package (SPSS; Armonk, NY, USA) version 26.0. The data was nonparametric since it was not normally distributed. Pearson's chi-square test was performed to analyze the perception of EFS during endodontic procedures between male and female DHOs from Pakistan. A *p* value at <0.05 was considered significant.

## 3. Results

A total of 800 DHOs participated in this study, of which 460 were females and 340 were males. The most common age group was between 24 and 26 years old. This questionnaire evaluated the perception of DHOs regarding separation of endodontic files during endodontic treatment.

**Figure 2:**
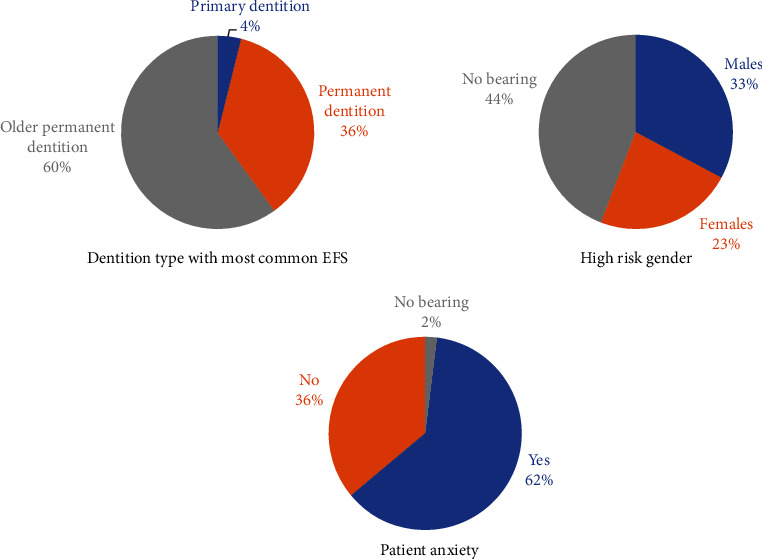
DHOs' perception for file separation during endodontic treatment in relation to patient-related factors. Data shown as a percentage of respondents (*n* = 800) (*p* value < 0.001).

**Figure 3:**
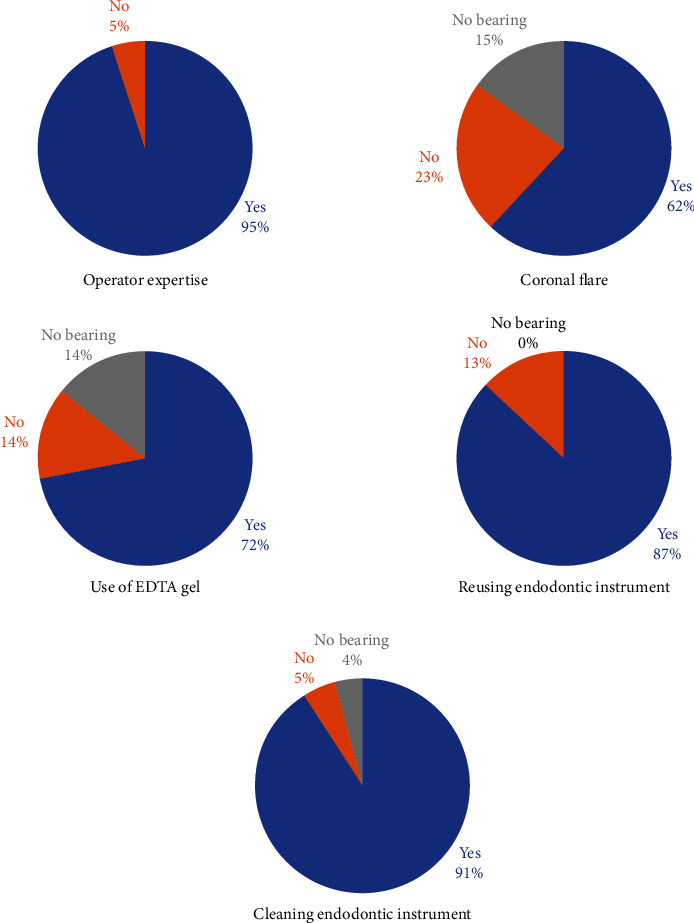
DHOs' perception for file separation during endodontic treatment in relation to operator-related factors. Data shown as a percentage of respondents (*n* = 800) (*p* value < 0.001).

The unavoidable event of file separation during root canal treatment was divided into four major factors.  Figure 2 shows DHOs' perceptions of file separation in relation to patient-related factors such as the most commonly involved dentition, gender at higher risk, and patient anxiety. Endodontic instrument separation most commonly occurs in older permanent dentition, according to the majority of house officers. In terms of which gender was more at risk of EFS, 43.7% said it happens regardless of gender risk. Moreover, 62% of respondents perceived that a patient's anxiety during treatment would contribute to instrument separation.  Figure 3 depicts the perception of DHOs in relation to operator-related factors that may cause EFS. The majority of house officers in our study believed that the higher the operators' experience, the lower the chances of a mishap such as endodontic instrument separation. In terms of achieving coronal flare as a preventive measure, most DHOs (61.7%) considered it a preventive measure. Furthermore, the majority of DHOs agreed to use EDTA gel (71.7%) and clean the instrument after each use (91.1%) to prevent EFS. Moreover, 87.5% of the DHOs agreed on their perception of knowledge about file reuse, which eventually leads to file separation.

Table 1 depicts DHOs' perception of file separation in relation to tooth anatomy. In terms of the most common teeth involved in file separation, 61.5% (492) of the DHOs perceived the posterior teeth as the most commonly involved. On the basis of the most common sites within the root canal that are involved in file separation, the majority (50.5%) of the DHOs perceived the apical third as the most common. Based on which portion of the root canal had the worst prognosis in the treatment plan of retrieving a separated instrument, 48.5% (388) of the DHOs thought the apical third of the canal had the poorest prognosis.  Table 2 depicts the perception of house officers for file separation in relation to endodontic instrument-related factors. Consideration of the best choice of instrument in endodontics was deemed an essential step by a majority (61.5%) of the DHOs. In comparison to rotary files, 56.7% of the DHOs perceived hand files to be the best choice of instrument. On the basis of the best alloy to avoid instrument separation, 45.2% of the DHOs perceived stainless steel as the best alloy, while 44.5% perceived NiTi to be the alloy of choice. In terms of phases involved in file separation, the majority (54.5%) of the DHOs perceived cleaning and shaping to be a critical step, while a considerable number (45.5%) of DHOs considered canal negotiation critical in causing file separation during endodontic treatment.

## 4. Discussion

Questionnaire-based surveys among freshly graduated DHOs are essential to critically analyze and unfold issues regarding how dentistry is being taught and perceived for better delivery of knowledge and for the development of practical skills. The best possible way to master a technique is to prevent the occurrence of a mishap in the first place by making the right choice in terms of patient diagnosis and further treatment planning. We speculate that by correlating DHOs' perceptions of EFS with available treatment outcomes, we can not only help to modulate how clinical knowledge is taught but also assist them in mastering their skills for removing separated endodontic instruments. Assessing the knowledge, attitude, and perception (KAP) among young dentists and endodontists will help in identifying knowledge gaps and patient-related factors, which in turn will aid in planning, evaluating, and implementing better treatment options. In this study, we have demonstrated how newly graduated DHOs perceive and understand EFS entirely in terms of patient-related factors, operator-relator factors, tooth anatomy, and endodontic instruments. Based on our survey, we evaluated how well fresh graduates are capable of handling mishaps during endodontic procedures. The findings from our study can be generalized to all other freshly graduated DHOs from different countries, as our survey can serve as an evaluating tool.

In accordance with the previous literature, we found that the perception of knowledge in relation to patient-related factors indicated a higher prevalence of EFS in older permanent dentition, which might be due to the fact that the pulp size and volume gradually diminish over time and become narrower in the old permanent dentition [17]. Constricted pulpal space increases the contact point between the file and dentin surface, producing more friction and higher chances of file separation. This finding is in concordance with the previous studies, reporting that the incidence of EFS is highest among people aged 21–40 years of age, with the highest rate of file fracture in people aged 31–40 years of age as compared to the 20–30-year age group. This may be because of the increased presence of the “aging society” in the elderly patients [18, 19].

It was further demonstrated in our study that the majority of DHOs (350; 43.7%) perceived that the incidence of file separation is not gender-dependent, while some (262; 32.7%) considered that males are at higher risk of file separation. In contrast, several previous studies from the literature report a higher incidence of file separation in females as compared with males [3, 20]. A previous study by Patnana et al. reported that the incidence of file separation was higher in females (4.6%) as compared to males (3.6%) [18]. Though there is no clear evidence backing the gender-specific incidence of EFS, it is speculated that it may be due to the differences in the root canal morphology between the two genders [21].

Our study demonstrated that the majority of the DHOs (492; 61.5%) perceive that posterior teeth are the most common site for file separation, possibly because of their perception that instrument separation may occur in posterior teeth, especially molars [12, 22]. Moreover, it is observed that young graduates believe treating posterior teeth is more difficult. According to Alrahabi, dental students were more confident in treating the anterior teeth. This is because the majority of the students (78%) performed their first endodontic treatment on the upper incisor and described it to be not difficult [23]. Furthermore, large proportion of clinical dentists (96.2%) including endodontists and general practitioners perceive that instruments usually break in molars [24]. Other reasons include the difficulty of treating multicanals, as DHOs typically treat single-canal teeth more during their undergraduate training than multiple canal teeth (molars) [25].

Among most of the incidents reported on instrument separation in molars, 39.5% of the separations occurred in the mesiobuccal canals [13]. Aside from the position of a tooth within the oral cavity, file separation within a canal is also significant. Many previous studies have identified the apical third of the canal as the most common site for file separation [14–16]. A retrospective clinical study previously revealed that fractures in the apical third are 33 times and six times more common than fractures in the middle third and coronal third portions of the root canal, respectively [21]. Madarati et al. showed that the risk of EFS in the apical third of the canal is higher than that in the coronal and middle thirds because most curved canals are narrow and the contact surface with the dentinal walls rises with curvature, causing file fatigue and separation [4]. Similarly, according to our study, 50.5% (404) of the house officers perceived the apical third to be the commonest site for endodontic file separation, while 26% (208) of the DHOs perceived it to be the middle third, and 23.5% of the house officers perceived it to be the coronal third. Considering all the factors, file separation treatment largely depends on the location of the file fracture in the canal. In concordance, a questionnaire-based study by Shilpa-Jain et al. revealed that the overall endodontists and postgraduates agreed that the location of the fractured instrument in the canal can affect instrument retrieval; 39.9% strongly agreed, while 53% agreed that the location of the instrument separation affects its retrieval [24].

In this study, 48.5% (388) of DHOs also believed that the apical third was the site with the poorest prognosis, while 33.5% (268) thought it was the middle third, and 18% (144) of DHOs perceived it to be the coronal third. The first and foremost step in the retrieval of the separated instrument is to identify the location of a broken piece within the canal and weigh the risks and benefits of removal of the separated instrument. According to medicolegal considerations, the patient needs to be informed about the inevitable incident of instrument separation, reassured, and guided through the new treatment plan. Approximately 82.9% of the participants agreed to notify the patient if an instrument was broken during the root canal procedure [24]. Both *in vivo* and *in vitro* studies from previous literature have shown that fractures localized in the apical third have the poorest prognosis [4, 26]. A recent study reported that a majority of the respondents (79.1%) reported a poor success rate in the removal of fractured instruments from the apical third in comparison with the middle third and coronal third [27]. File retrieval from the apical third increases the risk of a perforation and weakens the tooth structure due to excessive dentin removal, as well as increases the chances of ledge formation and extrusion of separated instruments beyond the apex [28]. The majority of the endodontists (64.8%) also presented a poor success rate in the removal of file instruments from the apical third and tended to bypass (66%) or leave (34%) the instruments fractured in the apical part of the root canal [27]. These findings assert that file retrieval from the apical third is challenging and requires adequate experience.

The majority of the respondents (592; 74%) in this study perceived that a good choice of instrument would most probably prevent instrument separation. Recent advancements have improved the properties of endodontic files in order to reduce the chances of endodontic complications. Some of the main file properties addressed in the present study were flexibility, strength, and fracture resistance. A questionnaire-based study of clinical dentists revealed that NiTi rotary was the most commonly fractured file (60.9%), followed by SS (12.7%), and NiTi hand file (3.2%) [24]. The same study reported that 21.9% of participants found multiple usage of files to be the most common reason for file fracture. Using NiTi files multiple times causes surface corrosion and imperfections, increasing the chances of file separation [29]. Adopting a single-use policy seems appropriate to avoid such clinical mishaps [30]. Furthermore, binding (53%) and ledge formation (45%) were the most common procedural problems encountered while attempting to remove or bypass the file [31]. A recent study compared the properties of SS files with NiTi rotary files for canal preparation. NiTi showed superiority in time consumption and canal shaping but also showed an increased incidence of file fracture as compared to SS. As a result, selecting the appropriate instrument for endodontic preparation of a tooth canal saves time and minimizes endodontic complications [7].

For quite some time, the advantages of rotary and manual filing systems have been debated. The file separation rates in both systems are highly debatable. About 56.7% (545) of DHOs in our study believed that manual files most frequently fracture in the canal as compared to rotary files. Some previous studies have shown that no significant difference occurs in file separation using rotary or manual canal preparation, except when an operator is experienced or inexperienced [8, 9]. On the contrary, in another study, the fracture rate reported with NiTi rotary was 5.6% as compared to that with manual stainless steel, which was 1.1% [7]. Another study reported that the odds of file separation with the rotary are seven times greater than that with hand instrumentation [6]. A retrospective study has reported that file separation incidence with the hand and rotary was found to be 0.25% and 1.68%, respectively [21]. This debate also includes what type of alloy is to be used for canal preparation with negligible risks and maximum output. Two commonly used alloys in endodontics are SS and NiTi. Generally, literature reports the same incidence of file fracture with NiTi files and SS files [10]. However, another study has reported NiTi files to be three times stronger, more flexible, and torsional fracture resistant as compared to SS files [4].

In this study, 45.2% (362) of the participants believed SS as a choice of alloy for endodontic procedures, while 44.5% (356) believed NiTi to be the choice of instrument. These findings are in contrast with a previous survey-based study among Swiss dentists, which reported that 80% of them preferred and integrated rotary NiTi into their practices [32]. However, several practitioners prefer the use of SS and NiTi hand files [33]. With their own advantages and disadvantages, it is evident that the choice of material is debatable and depends on the dentist's preference [34, 35].

Many factors contribute to the incidence of EFS. Some studies suggest file separation occurs more often in the early stages of canal preparation due to the smaller diameter, which in turn leads to an increase in torsional forces. Other studies suggest that file separation in later stages of canal preparation is more common due to the use of larger and stiffer files [10]. In our study, 54.4% (436) of the DHOs believed that most cases of file separation occur during cleaning and shaping of the canal, while according to 45.5% (364) of the house officers, file separation occurs frequently during negotiation of the canals. Similar results were reported from the previous survey-based study by Avoaka-Boni et al. reporting that the majority (60.5%) of them thought EFS occurred during cleaning and shaping [36].

Previous studies state that dentists require maximum training and comprehensive knowledge regarding endodontic instrumentation and required techniques for EFS retrieval [26]. A number of factors contribute to EFS such as the number of times an instrument is used, as well as operators' skill and level of expertise. Overall, 95.2% (762) of DHOs in our study agreed that the operators' expertise was equally essential to prevent any endodontic complication and in providing the best prognosis to a decaying tooth. In accordance to our findings, a survey-based study by Madarati et al. reported that 54.6% of endodontists and general practitioners felt that factors related to the operator were the most important aspects that contributed to EFS [37].

Preventive measures should be taken in order to reduce the risk of EFS. A coronal flare is provided to achieve straight-line access. This reduces the two-point flexure on the file and provides a better glide path. Studies have proven a reduced rate of file separation when coronal flare is provided [38, 39]. It is also stated that coronal flaring reduces torsional stress as the canal width becomes almost equal to the diameter of the instrument, which also provides an understanding of the canal anatomy [4, 40]. From proper canal preparation technique to adequate irrigation, EDTA use, and cleaning of endodontic instruments before use, all are considered essential [41]. Calcium-chelating agents such as EDTA soften the dentin walls by forming stable calcium complexes with nonorganic components of dentin. The use of such lubricants has been previously demonstrated to reduce the risk of file fractures [42, 43]. Hence, emphasis on cleaning of endodontic instruments prior to use in order to prevent any mishap is essential [44].

Canal patency is maintained by recapitulating the small-size stainless steel K-file through the apical foramen or beyond the existing working length to remove any debris being accumulated during dentin cutting [45]. Thus, this procedure ensures biological cleansing of the apical foramen for irrigants to flow and prevent dislodgement of unwanted debris into the curvatures of the file that creates friction [46]. A glide path ensures regular opening from the coronal part to the apex. It has been shown in previous studies that an effective glide path prevents file fractures when high forces are used in narrow canals [47]. Moreover, it has also been demonstrated that an effective glide path reduces torsional forces and increases the life of rotary NiTi files by almost six times [48]. Similarly, lubricants such as sodium hypochlorite and EDTA rinse out debris and reduce friction between files and narrow canals [49].

In accordance with previous literature, 91% (728) of the DHOs in our study also perceived that proper cleaning of files after every usage is also essential. In this study, 61.7% (494) of the house officers believe that coronal flare reduces the chances of file separation, and 22.7% (182) of the house officers do not consider coronal flaring a preventive measure in reducing the chances of file separation. In reference to the use of EDTA, 71.7% (574) of the house officers in our study believe that use of EDTA is a good preventive measure, while 14% (112) of the house officers do not consider it as one of the preventive measures, and 91% (728) of the participants perceive that proper cleaning of files after every usage is also essential. Lunelli et al. investigated the cleaning, sterilization, and storage processes of endodontic instruments through a questionnaire-based study among endodontists. The brush-associated cleaning process using enzymatic detergent was adopted by most of the participants. The instruments were sterilized using an autoclave. About 49% used the instruments till they had twisted or fractured, while 51% disposed of them based on the number of uses, with a mean age of five uses for rotary instruments [50].

The majority of the DHOs (700; 87.5%) perceived that repeated use of endodontic instruments subjected them to fractures. Repeated use of files subjects them to repeated autoclave cycles. An increase in the number of autoclave cycles increases the surface roughness of files and thus increases the chances of file breakage [51]. However, Patturaja et al. reported that the majority of the dental specialists (88%) prefer cleaning their rotary instruments for multiple uses, with most of them (54.1%) cleaning at least three times a week [52].

Despite the fact that this questionnaire-based study provides insights into DHOs' opinions and attitudes toward EFS, it has some limitations due to its cross-sectional nature, which might involve chances of bias. A better understanding of EFS among young DHOs would undoubtedly boost their confidence in managing file separation during treatment. However, the management of such clinical mishaps varies depending on the situation and the patient's choice. Furthermore, it would be interesting to study the perception of these young DHOs as to how they would tackle the EFS in their future clinical experience. Though our questionnaire revealed that the majority of the DHOs perceive choice of instrument to prevent EFS, multiple usage of files as a reason of fracture, SS to be the choice of alloy, and operator's expertise as well as cleaning of files to be essential, more research is needed into the DHOs' choices and decisions when approaching the EFS treatment plan.

## 5. Conclusion

This study clearly demonstrated that the DHOs were aware of and understood most of the aspects related to file separation during endodontic treatment. The majority of DHOs believe that EFS is more vulnerable in mesiobuccal canals of the apical third of posterior teeth during root canal shaping and cleaning. Moreover, most of them thought that SS was a better choice of alloy for file instruments. Manual files over rotary files were believed to be more prone to fractures due to repeated use. Proper cleaning with coronal flares and EDTA was perceived to reduce the chances of file separation.

## Figures and Tables

**Table 1:** House officer perception for file separation during endodontic treatments in relation to tooth anatomy.

Factors related to anatomy	Number of responses			Total respondents	*p* value^∗^
Location of tooth	Anterior	Posterior	No bearing	800	3.55*E* − 83
	266 (33.2)	492 (61.5)	42 (5.2)		
Portion of root	Coronal	Middle	Apical		6.30*E* − 24
	188 (23.5)	208 (26)	404 (50.5)		
Poorest prognosis	Coronal	Middle	Apical		5.72*E* − 25
	144 (18)	268 (33.5)	388 (48.5)		

**Table 2:** House officer perception for file separation during endodontic treatment in relation to endodontic instrument factors.

Endodontic instrument-related factors	Number of responses			Total respondents	*p* value^∗^
Choice of endodontic instrument	Yes	No	No bearing	800	6.86*E* − 132
	592 (74)	138 (17.2)	70 (8.7)		
Type of endodontic file	Rotary	Hand file	No bearing		6.24*E* − 88
	338 (42.2)	454 (56.7)	8 (1)		
Endodontic instrument “alloy”	Stainless steel	NiTi	No bearing		2.14*E* − 42
	362 (45.2)	356 (44.5)	82 (10.2)		
Phase of “root canal treatment”	While negotiating the canal	During cleaning and shaping	No bearing		0.011
	364 (45.5)	436 (54.5)	0		

## Data Availability

The dataset used in the current study will be made available at the reasonable request.
