# Supplementary material for: Perception of Dental House Officers regarding Endodontic File Separation during Endodontic Treatment
Source: Biomed Res Int. 2023 Feb 16;2023:1044541. doi: 10.1155/2023/1044541 (PMC9949941; doi:10.1155/2023/1044541)
Supplement: Supplementary Materials — The questionnaire used in the current study is provided as a supplementary file. [file 1044541.f1.pdf]

Evaluation of Knowledge of House Officers Regarding Prevention of Endodontic Instrument Fracture during Endodontic Treatment

|QUESTIONNAIRE|

Your participation in this study will be highly appreciated. Please answer all 15 questions by circling the appropriate answer of your choice where required. **All your information will be kept confidential and used only for research and educational purpose.**

Gender: M/F **Age: ____**

College / Institution: ____

Year of Graduation: ________

1) **In which of the following is endodontic instrument fracture more common, in your opinion?**
   a) **Primary dentition**
   b) **Young permanent teeth**
   c) **Old permanent teeth**
2) **In your opinion ,which gender is at a higher risk of endodontic instrument fracture :**
   a) **Males**
   b) **Females**
   c) **No bearing**
3) **In your opinion, which of the following location of a tooth can be a contributory factor for endodontic instrument fracture?**
   a) **Anterior**
   b) **Posterior**
   c) **No bearing**
4) **Patient anxiety plays a role in endodontic instrument fracture :**
   a) **True**
   b) **False**
5) **Which portion of the root canal is more prone to instrument fracture?**
   a) **Coronal Third**
   b) **Middle Third**
   c) **Apical Third**

6) **Does Expertise of the operator plays a role in the prevention of endodontic instrument fracture :**
   a) **Yes**
   b) **No**

7) **Does "Coronal Flare" reduce the chances of endodontic instrument fracture?**
   a) **Yes**
   b) **No**
   c) **No bearing**
8) **Do you agree that use of "EDTA GEL" (glide) reduces risk of endodontic instrument fracture?**
   a) **Yes**
   b) **No**
   c) **No bearing**
9) **Does cleaning of an endodontic instrument, during instrumentation of root canal, help in prevention of its fracture?**
   a) **Yes**
   b) **No**
   c) **No bearing**
10) **Does reusing of used endodontic instrument, increases the risk of instrument fracture?**
   a) **Yes**

b) **No**
c) **No bearing**

11) **Does the choice of endodontic instrument reduce the risk of instrument fracture?**
    a) **Yes**
    b) **No**
    c) **No bearing**
12) **In your opinion , which type of endodontic file , frequently breaks in the root canal :**
    a) **Rotary files**
    b) **Hand files**
    c) **No bearing**
13) **In your opinion, which type of endodontic instrument “alloy” is more frequently broken?**
    a) **Stainless Steel**
    b) **Nickel Titanium**
    c) **No bearing**
14) **In your opinion, in which phase of “Root Canal Treatment”, instruments are most likely broken?**
    a) **While negotiating the canal**
    b) **During cleaning and shaping**
    c) **None of the above**
15) **In your opinion, which of the following root canal area has the poorest prognosis of fractured instrument retrieval?**
    a) **Coronal Third**
    b) **Middle Third**
    c) **Apical Third**
